# Trends and Risk Factors for Surgical Site Infection after Treatment of the Ankle Fracture: National Cohort Study

**DOI:** 10.3390/jcm12134215

**Published:** 2023-06-22

**Authors:** Hwa-Jun Kang, Young-Min Kwon, Sun-ju Byeon, Hyong Nyun Kim, Il-Hoon Sung, Sivakumar Allur Subramanian, Sung Jae Kim

**Affiliations:** 1 Department of Orthopedic Surgery, Hallym University Dongtan Sacred Heart Hospital, Hwaseong 18450, Republic of Korea; ospigy@naver.com (H.-J.K.); kwonym0814@gmail.com (Y.-M.K.); drsivaphdbio@gmail.com (S.A.S.); 2 Department of Pathology, Yuseong Sun Hospital, Daejeon 34084, Republic of Korea; meilleur@meilleursolution.com; 3 Department of Orthopedic Surgery, Hallym University Kangnam Sacred Heart Hospital, Seoul 07441, Republic of Korea; hyongnyun@naver.com; 4 Department of Orthopedic Surgery, Hanyang University Hospital, Seoul 04763, Republic of Korea; sungih@hanyang.ac.kr

**Keywords:** surgical site infection, ankle fracture, national cohort study

## Abstract

Surgical site infection (SSI) is a major complication after the surgical treatment of ankle fractures that can result in catastrophic consequences. This study aimed to determine the incidence of SSI in several cohorts from national insurance databases over the past 12 years and identify its predictors. The claimed data for patients (*n* = 1,449,692) with ankle fractures between 2007 and 2019 were investigated, and a total of 41,071 patients were included in the final analysis. The covariates included were age, sex, season, fracture type (closed vs. open), type of surgical fixation procedure, and comorbidities of each patient. All subjects were divided into two groups according to the SSI after the surgical fixation of the ankle fracture (no infection group vs. infection group). The number of SSIs after the surgical treatment of ankle fractures was 874 (2.13%). Open fractures [odds ratio, (OR) = 4.220] showed the highest risk for SSI, followed by the male sex (OR = 1.841), an increasing number of comorbidities (3–5, OR = 1.484; ≥6, OR = 1.730), a history of dementia (OR = 1.720) or of myocardial infarction (OR = 1.628), and increasing age (OR = 1.010). The summer season (OR = 1.349) showed the highest risk among the four seasons for SSI after ankle fracture surgery.

## 1. Introduction

Ankle fractures are the fifth most common fracture, accounting for 9% of all fractures [1,2,3]. It is an extremely common injury encountered in practice, and the incidence is reported to be 184 per 100,000 person-years [4]. The incidence of ankle fractures increases with age [1,2,3].

Surgical site infection (SSI) is a major complication after the surgical treatment of ankle fractures, with rates ranging from 1% to 8% in previous studies [5]. These infections can result in catastrophic consequences, such as impaired joint function, joint stiffness, arthritis, limb loss, or even death [6,7]. A previous study reported that even after successfully eradicating the infection, the functional outcomes were poor 3–6 years after surgery in patients with SSI [8].

Previous studies have examined the incidence of SSI after the surgical treatment of ankle fractures and identified its risk factors. However, the criteria used for the diagnosis of SSI were inconsistent among studies, and the number of patients investigated was relatively small in most studies.

We investigated SSI after the surgical treatment of ankle fractures in 41,071 patients identified in the national health care database. This study aimed to determine the incidence of SSI in several cohorts over the past 12 years, identify its predictors, including seasonal variation, and identify age-specific risk factors for SSI.

## 2. Materials and Methods

### 2.1. Dataset

This study was approved by the Institutional Review Board of our institute (HUDH 20 February 2020). This was a retrospective cohort study using claims data from the Health Insurance Review and Assessment, which oversees the payment review of the National Health Insurance Sharing Service, wherein more than 97% of Koreans are enrolled.

### 2.2. Study Design and Participants

We extracted the claims data for patients (*n* = 1,449,692) with diagnostic codes for ankle fractures (medial malleolar, lateral malleolar, bimalleolar, and trimalleolar fractures) between 2007 and 2019.

**Figure 1:**
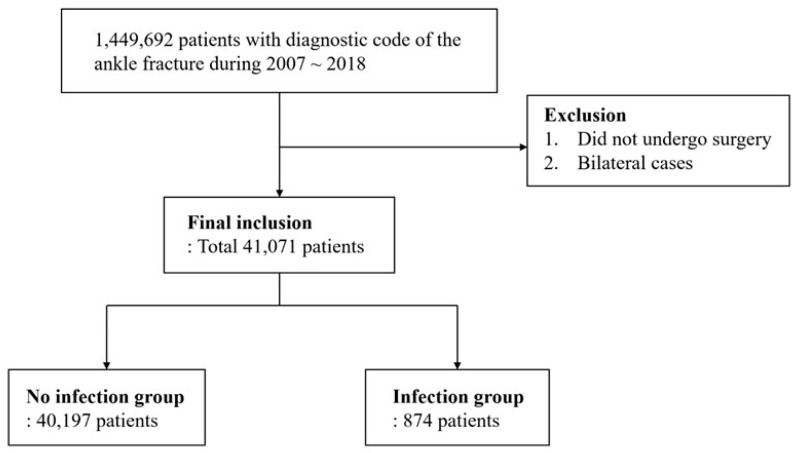
Study design using claims data from the Health Insurance Review and Assessment.

Among these patients, those who did not undergo surgical fixation procedures were excluded. Patients who suffered an injury and underwent surgical fixation in 2019 and were not followed up for at least 1 year after surgery were excluded. As bilaterality could not be identified in the current database system, we excluded cases of bilateral ankle fractures. To exclude bilateral fractures, we excluded cases with surgical fracture fixation procedures performed more than once during the study period (Figure 1).

### 2.3. Definition of the SSI

In clinical settings, SSI after fracture fixation is usually treated with serial irrigation and debridement procedures (I&D). However, cases in which bacterial infections are not able to be eradicated with serial I&D procedures progressed to the early fixation device removal procedures for eradication of the bacterial biofilms formed on the surface of the implant and surrounding tissues. Finally, in some cases with progressed complicated bone infection after SSI, bone curettage procedures are needed for the eradication of bacterial infection.

Therefore, the SSI was defined as the case followed by additional bacterial eradicating procedure codes found in our databases after initial surgical treatment for ankle fractures. The additional bacterial eradicating surgical procedures were defined as follows: (1) I&D procedures, (2) early removal of the surgical fixation device for surgical fracture fixation followed by serial I&D procedure, or (3) additional bone curettage procedures. Early removal of the fixation device was defined as the removal of the fixation device earlier than 3 months following initial ankle fracture surgical fixation procedures based on the usual clinical scenario. In usual clinical settings, most of the fixation device removal procedures were carried out because of patients’ desires or for prevention of possible stress shield effects of surrounding bone tissues by the fixation device with titanium plate after fracture healing. Therefore, the fixation device removal procedures performed more than three months after initial surgical fixation procedures for the ankle fractures, or not followed by serial I&D procedures, were not considered as additional bacterial eradicating procedures for SSI.

### 2.4. Covariates

The covariates included age, sex, season, fracture type (closed vs. open), type of the initial surgical treatment procedure (closed reduction and internal fixation vs. open reduction and internal fixation), and comorbidities of each patient. The included comorbidities were selected from the Charlson comorbidity index (CCI). All the variables were recorded on a dichotomous scale except age (continuous variable) and season (four scales: spring, summer, autumn, and winter), hospital grade (four scales: private clinic, hospital, general hospital, and tertiary general hospital), CCI score (three scales: <3 comorbidities, 3–5 comorbidities, and >6 comorbidities).

### 2.5. Statistical Analysis

All subjects were divided into two groups according to the SSI after the surgical fixation of the ankle fracture (no infection group vs. infection group). We used Pearson’s chi-square test to compare the differences in covariates between the two groups. An independent samples *t*-test was used to compare the ages of the two groups. We analyzed covariates using binary logistic regression analysis to analyze the factors associated with SSI after ankle fracture. In the multivariate binary logistic regression analysis, a stepwise algorithm based on the Akaike information criterion was used to optimize the analysis model. Statistical significance was set at *p* < 0.05. All statistical analyses were performed using R v. 4.0.3 (R Core Team, http://www.R-project.org/, 15 June 2020).

## 3. Results

The demographic data of the patients included in the current study are summarized in Table 1. A total of 41,071 patients were included. The mean age was 47.3 ± 18.64 years. The number of SSIs after the surgical treatment of ankle fractures was 874 (2.13%). The mean time of SSI diagnosis after ankle fracture surgery was 35.8 ± 28.02 days. Among these patients with SSI, 744 (85.1%) were treated with only serial I&D procedures, and 93 (10.6%) required additional early plate removal.

The results of the univariate comparison analysis of the two groups are summarized in Table 2. The infection group comprised significantly older individuals than the no infection group (49.7 ± 19.99 vs. 47.2 ± 18.61, *p* < 0.001). The infection group had a significantly higher number of patients with open fractures (18.1% vs. 4.9%, *p* < 0.001).

The results of the multiple logistic regression analysis are summarized in Table 3. During the adjusted odds ratio (OR) calculation, a significantly higher OR was observed in the summer than in the spring (OR = 1.349, *p* = 0.003). A trend of lower OR compared with spring was observed in the winter; however, we could not find statistical significance in the current study. Males showed a significantly higher OR for SSI (OR = 1.841, *p* < 0.001) compared with females. Open fractures showed a significantly higher risk for SSI (OR = 4.220, *p* < 0.001). As the number of comorbidities increased, the risk of SSI also significantly increased (CCI of 3–5, OR = 1.484; CCI of >6, OR = 1.730; *p* < 0.001 for both).

**Figure 2:**
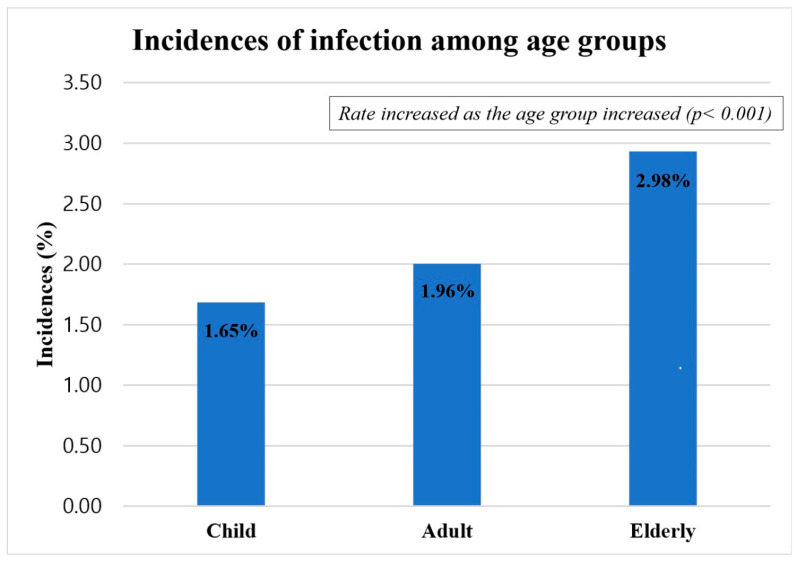
Incidence of surgical site infection after ankle fracture surgery among age groups.

The incidence of SSI after the surgical treatment of ankle fractures based on the age group is depicted in Figure 2. The infection rate among children was 1.65% (39/2319), among adults was 1.96% (614/30684), and among the elderly was 2.98% (221/7194), respectively. The incidence rate based on the age group showed significantly different results (*p* < 0.001). The Cochran–Armitage test for trend analysis also showed that the incidence rate significantly increased as the age of the group increased (*p* < 0.001).

The results of the multiple logistic regression analysis stratified by the age group are summarized in Table 4. Among children, only open fractures showed a significant risk (OR = 4.804, *p* = 0.001). Among adults, open fractures showed the greatest OR for SSI (OR = 4.837, *p* < 0.001). Interestingly, a significant risk (OR = 1.350, *p* = 0.012) for SSI among adults was observed in the summer. Male sex, a history of cerebrovascular disease, and chronic pulmonary disease were significant risk factors for SSI. Among the elderly, open fractures showed the highest risk for SSI (OR = 2.570, *p* < 0.011). Male sex, increasing age, and a history of liver disease showed significant ORs for SSI among the elderly.

## 4. Discussion

Infection after surgical treatment of fractures burdens the patient and the social health care system. The increased treatment cost per patient is almost seven times higher than that in uninfected cases, and a prolonged hospital stay, with a median of 2 weeks longer, is the most important factor [9,10,11,12]. Korim et al. evaluated 710 cases of ankle fractures treated with open reduction and internal fixation techniques and noted that SSI significantly reduced the patient outcome score (60 vs. 90, Olerud–Molander Ankle Score) [13]. A recent systematic review also revealed that reinfection occurs in 6–9% of the cases, and amputation of the affected limb was required in 3–5% of the cases [14]. Periprosthetic infection can cause biofilm formation on implants and necessitate implant removal to eradicate infection before bone union can be achieved [6]. Therefore, prevention is the best option for SSIs after treating ankle fractures. Previous studies recommended that antibiotic prophylaxis should be administered within 60 min before surgery [15] and should be fully injected before tourniquet inflation [16].

The incidence of SSI after the surgical treatment of ankle fractures was 2.13% in the current study. Significant predictors of SSI were the summer season (OR = 1.349, *p* = 0.003), male sex (OR = 1.841, *p* < 0.001), increasing age (OR = 1.010, *p* < 0.001), open fractures (OR = 4.220, *p* < 0.001), myocardial infarction (OR = 1.628, *p* = 0.038), and dementia (OR = 1.720, *p* = 0.017).

Previous studies have reported various incidences of SSI after the surgical treatment of ankle fractures. The current study has the advantage of being one of the largest cohorts of patients with ankle fractures. Previous studies with 1000 or more cases revealed that the incidence of SSI after the surgical treatment of ankle fractures was 5.7–6.8% [4,17,18]. A prior study with the largest cohort of patients with ankle fractures was also used in a hospital discharge database, and it included 57,183 patients with ankle fractures. The incidence of SSI after ankle fracture in their study was 1.44% [6], similar to our study. The reason for the inconsistent incidence of SSI after ankle fracture surgery among studies seems to lie in the inconsistent definition of SSI after surgical treatment.

Several risk factors have been suggested for SSIs after the treatment of ankle fractures. Certain previous studies reported that smoking, obesity, alcohol consumption, and prolonged operative time (>90 min) are mostly related to SSI in periarticular ankle fractures [17,18,19,20]. Diabetes, open fractures, and peripheral vascular disease have also been suggested as significant risk factors for SSI 6. Another study reported that electrolyte imbalance and coagulopathies can also be independent risk factors for SSI [21]. Advanced age, male sex, lower albumin levels, and chronic heart disease were also noted as non-modifiable risk factors for SSI after the treatment of ankle fractures [7,22]. Some studies also have suggested that Weber C fractures (OR = 4.0, *p* = 0.048) were significant risk factors for SSI [13]. This may be supported by the results of previous experimental studies showing that an unstable fracture lowers the host's defense mechanism against infection [12].

Interestingly, the current study revealed that the summer season showed significantly high OR for SSI after the treatment of ankle fractures. Relatively humid and hot weather of the summer season may cause difficulties in maintaining cleanliness around surgical incisions within walker braces or splints after surgical treatment. A previous study also reported that the cleanliness of the surgical incision is a significant risk factor for SSI [3]. Similar to previous studies, male patients showed a significantly higher OR (1.841) than female patients in the current study. This may be related to the high possibility of smoking and low personal hygiene.

Our study did not identify diabetes as a risk factor for SSI after the treatment of ankle fractures. We tried several statistical techniques such as propensity score matching and making subgroups analysis with complicated diabetes groups; however, no model revealed the statistical significance of diabetes as a risk factor for SSI. A previous study also demonstrated that diabetes was not significantly correlated with infection [23]. This may be because the surgical incision for treatment of the ankle fracture is located at the level of the ankle joint, not the distal foot area, where diabetic angiopathy is more related to wound healing. However, further studies on the relationship between diabetes and SSI after ankle fracture surgery are needed for a definite conclusion.

Similar to previous studies, increasing age, open fractures, and heart diseases such as myocardial infarction showed a significantly high OR for SSI in the current study. Notably, patients with dementia showed high odds for SSI (OR = 1.720) in the current study. Patients with dementia usually have difficulties keeping up with independent daily activities and good general hygiene, which may be related to an increased risk of wound infection.

The current study has several limitations. First, we defined SSI after the surgical treatment of ankle fracture based on the appropriate clinical scenario of SSI. This could result in an underestimation of SSI. However, our incidence of SSI after the treatment of ankle fracture corroborates previous studies, and most of the statistical analyses performed in the current study showed high significance. The second, obesity and smoking history, may have an important relationship with SSI, but we could not have used them as independent factors in the statistical analysis. Only patients who visited the clinic for treating obesity were defined as obese individuals. Body mass index (BMI) is a better selection for analyzing the effect of obesity on SSI. However, BMI and smoking history were not included in the database platform used for the current analysis. Third, we could not include the fracture severity type as an independent variable in a statistical model, because the database we used for the current analysis was a national cohort for insurance services and did not include a detailed description of fracture severity type. However, we believe the advantage of a large cohort and high statistical significance for some of the risk factors found in the current study still have some importance. In addition, our statistical analysis showed that the larger the size of the hospital, the higher incidences of the SSI. However, in real clinical situations, severely comminuted fractures or complicated open fractures are usually more transferred to general or tertiary-sized hospitals. Due to the limitation of not including the fracture severity type in the statistical model, the current analysis for the relation between this SSI and the hospital grade also has a risk of selection bias. Fourth, although previous studies noted the importance of antibiotic prophylaxis within 60 min before surgery [15], and before tourniquet inflation [16], we could not include the timing of prophylactic antibiotic use as an independent variable in the current statistical model. However, we think that the great number of subjects included in the current study, and the high statistical significances for other risk factors found in the current model still have valuable clinically relevant meanings.

## 5. Conclusions

In conclusion, the incidence rate of SSI after the surgical treatment of 41,071 patients with ankle fractures was 2.13% in our study. Open fractures (OR = 4.220) showed the highest risk for developing SSI, followed by the male sex, an increasing number of comorbidities, a history of dementia, a history of myocardial infarction, and increasing age. Seasons also seem to be related to SSI; the summer season showed the highest risk for SSI after ankle fracture surgery.

## Figures and Tables

**Table 1:** Demographic data of patients.

Number of Patients	41,071
Age (years)	47.3 ± 18.64
Sex (male:female)	22,064:19,007
Hospital day (day)	16.7 ± 12.69
Fracture type (closed:open)	38,942:2129
Fixation type (CRIF:ORIF) *	1220:39,851
Hospital grade (A/B/C/D) **	2870/15,155/16,368/6678
Periprosthetic infection (*n*, %)	874 (2.13%)
Mean time of SSI *** (days, mean, SD)	35.8 ± 28.02
Treatment for SSI (A:B:C) ****	744 (85.1%):93 (10.6%):37 (4.3%)

* CRIF, closed reduction and internal fixation; ORIF, open reduction and internal fixation. ** A, clinic; B, hospital; C, general hospital; D, tertiary hospital; *** SSI, surgical site infection. **** A, treated with irrigation and soft tissue debridement; B, treated with including metal removal; C, treated with including infected bone curettage.

**Table 2:** Comparison between two groups.

	No Infection	Infection	*p* Value
Number of patients	40,197	874	
Age (mean, standard deviation)	47.2 ± 18.61	49.7 ± 19.99	**<0.001**
Sex (male: female)	21,484:18,713 (53.4%: 46.6%)	580:294 (66.4%: 33.6%)	**<0.001**
Admission duration (day)	17.3 ± 11.27	16.4 ± 10.3	0.300
Season			**<0.001**
Spring	9420	191	
Summer	8486	231	
Fall	9658	238	
Winter	12633	214	
Fracture type (closed/open) *	38,226/1971 (95.1%: 4.9%)	716/158 (81.9%: 18.1%)	**<0.001**
Fixation type (CRIF/ORIF) **	1195/39,002	25/849	0.926
Hospital grade (A/B/C/D)	2827/14,899/15,994/6477 (7.0%/37.1%/39.8%/16.1%)	43/256/374/201 (4.9%/29.3%/42.8%/23.0%)	**<0.001**
Comorbidities (*n*, %)			
Myocardial infarction	484 (1.2%)	21 (2.4%)	**0.003**
Congestive heart failure	1091 (2.7%)	32 (3.7%)	0.111
Peripheral vascular disease	3839 (9.6%)	80 (9.2%)	0.736
Cerebrovascular disease	2053 (5.1%)	65 (7.4%)	**0.003**
Dementia	522 (1.3%)	23 (2.6%)	**0.001**
Chronic pulmonary disease	8048 (20.0%)	187 (21.4%)	0.336
Connective tissue disease	2668 (6.6%)	52 (5.9%)	0.459
Peptic ulcer	8505 (21.2%)	197 (22.5%)	0.344
Liver disease	6704 (16.7%)	161 (18.4%)	0.187
Diabetes	3857 (9.6%)	93 (10.6%)	0.328
Hemiplegia	508 (1.3%)	19 (2.2%)	**0.027**
Cancer (no metastasis)	921 (2.3%)	26 (3.0%)	0.223
Cancer (metastasis)	215 (0.5%)	8 (0.9%)	0.200
Acute renal failure	303 (0.8%)	10 (1.1%)	0.264
Chronic renal failure	51 (0.1%)	1 (0.1%)	1.000
Rheumatoid arthritis	390 (1.0%)	5 (0.6%)	0.309
Osteoporosis	2174 (5.4%)	47 (5.4%)	1.000
Hyperlipidemia	3677 (9.1%)	92 (10.5%)	0.181

* Close fracture/open fracture; ** CRIF, closed reduction and internal fixation; ORIF, open reduction and internal fixation. Bolds indicate significant value.

**Table 3:** Results of multiple logistic regression analysis for periprosthetic infection.

	Crude OR (95% CI)	*p* Value	Adjusted OR (95% CI)	*p* Value
Season				
Spring	Reference		Reference	
Summer	1.343 (1.106–1.631)	0.003	1.349 (1.110–1.641)	**0.003**
Autumn	1.215 (1.003–1.475)	0.047	1.221 (1.007–1.484)	**0.043**
Winter	0.835 (0.686–1.018)	0.074	0.850 (0.697–1.036)	0.107
Sex (female as reference)	1.718 (1.493–1.982)	<0.001	1.841 (1.590–2.136)	**<0.001**
Age	1.007 (1.004–1.011)	<0.001	1.010 (1.006–1.014)	**<0.001**
Fixation type (CRIF vs. ORIF)	1.041 (0.712–1.596)	0.846	0.945 (0.645–1.453)	0.783
Open fracture	4.280 (3.51–5.100)	<0.001	4.220 (3.515–5.038)	**<0.001**
Comorbidities				
Myocardial infarction	2.020 (1.259–3.061)	0.002	1.628 (0.998–2.516)	**0.038**
Congestive heart failure	1.362 (0.933–1.915)	0.091	1.184 (0.799–1.694)	0.377
Peripheral vascular disease	0.954 (0.751–1.196)	0.693	0.823 (0.638–1.049)	0.124
Cerebrovascular disease	1.493 (1.144–1.913)	0.002	1.274 (0.957–1.671)	0.088
Dementia	2.054 (1.308–3.061)	0.001	1.720 (1.074–2.628)	**0.017**
Chronic pulmonary disease	1.087 (0.921–1.277)	0.315	1.147 (0.965–1.358)	0.114
Connective tissue disease	0.890 (0.663–1.168)	0.419	0.862 (0.635–1.145)	0.322
Peptic ulcer	1.084 (0.921–1.270)	0.323	1.034 (0.871–1.222)	0.702
Liver disease	1.128 (0.946–1.337)	0.172	1.057 (0.874–1.273)	0.561
Diabetes	1.122 (0.897–1.387)	0.300	0.956 (0.752–1.204)	0.709
Hemiplegia	1.736 (1.056–2.679)	0.020	1.313 (0.787–2.062)	0.266
Cancer (no metastasis)	1.308 (0.859–1.900)	0.184	1.036 (0.671–1.529)	0.867
Cancer (metastasis)	1.718 (0.775–3.261)	0.135	1.563 (0.695–3.033)	0.229
Acute renal failure	1.524 (0.755–2.717)	0.192	1.201 (0.587–2.184)	0.582
Chronic renal failure	0.902 (0.051–4.111)	0.918	0.575 (0.032–2.720)	0.588
Rheumatoid arthritis	0.587 (0.209–1.276)	0.238	0.531 (0.188–1.163)	0.164
Osteoporosis	0.994 (0.729–1.322)	0.968	0.944 (0.683–1.274)	0.716
Hyperlipidemia	1.168 (0.933–1.446)	0.163	1.099 (0.866–1.380)	0.426
Charlson comorbidity index				
0~2 (*n* = 27746)	Reference		Reference	
3–5 (*n* = 6499)	1.481 (1.189–1.840)	<0.001	1.484 (1.191–1.843)	**<0.001**
6- (*n* = 2047)	1.725 (1.258–2.340)	0.001	1.730 (1.261–2.345)	**<0.001**

OR, odds ratio; CRIF, closed reduction and internal fixation; ORIF, open reduction and internal fixation. Bolds indicate significant value.

**Table 4:** Result of logistic regression for periprosthetic infection stratified by age group.

	Odds Ratio (95% CI)	*p* Value
Child Group (*n* = 2358)		
Season		
Spring	Reference	
Summer	2.219 (0.960–5.397)	0.066
Autumn	1.281 (0.524–3.210)	0.586
Winter	0.623 (0.190–1.819)	0.400
Fixation type	6.190 (1.330–110.245)	0.073
Open fracture	4.804 (1.760–11.151)	**0.001**
Adult Group (*n* = 31,298)		
Season		
Spring	Reference	
Summer	1.350 (1.070–1.707)	**0.012**
Autumn	1.233 (0.981–1.554)	0.074
Winter	0.862 (0.681–1.093)	0.218
Sex	1.870 (1.570–2.237)	**<0.001**
Open fracture	4.837 (3.933–5.908)	**<0.001**
Cerebrovascular disease	1.550 (1.034–2.230)	**0.025**
Chronic pulmonary disease	1.297 (1.062–1.572)	**0.009**
Rheumatic arthritis	0.185 (0.011–0.827)	0.092
Elderly Group (*n* = 7415)		
Sex	1.921 (1.467–2.520)	**<0.001**
Age	1.042 (1.017–1.066)	**0.001**
Open fracture	2.570 (1.628–3.893)	**<0.001**
Myocardial infarction	1.694 (0.939–2.844)	0.060
Peripheral vascular disease	0.721 (0.499–1.019)	0.060
Dementia	1.578 (0.908–2.571)	0.084
Liver disease	1.365 (1.008–1.832)	**0.041**

Child group, 1~15 years; adult group, 16~65 years; elderly group, 66~ years; bolds indicate significant values.

## Data Availability

The data from this study are not publicly available because of the privacy and ethical restrictions of the Korean National Health Insurance data sharing system. The dataset used in this study can only be accessed by an authorized researcher.
